# The impact of gut microbiota on morbidities in preterm infants

**DOI:** 10.1002/kjm2.12878

**Published:** 2024-07-29

**Authors:** Mei‐Yin Lai, Yin‐Hsi Chang, Chien‐Chung Lee, Neonatal Microbiome Outcomes Study Group (NEMO), Cheng‐Hsun Chiu, Ming‐Chou Chiang, Wei‐Chi Wu, Yuan‐Ming Yeh, Wei‐Hung Wu, Po‐Yi Wu, Ming‐Chih Ho

**Affiliations:** ^1^ Division of Neonatology, Department of Pediatrics, Chang Gung Memorial Hospital, School of Medicine Chang Gung University Taoyuan Taiwan; ^2^ Department of Ophthalmology, Chang Gung Memorial Hospital, School of Medicine Chang Gung University Taoyuan Taiwan; ^3^ Chang Gung Memorial Hospital Taoyuan Taiwan

**Keywords:** dysbiosis, gut microbiota, gut–organ axis, morbidity, preterm

## Abstract

The gut microbiota undergoes substantial development from birth, and its development in the initial years of life has a potentially lifelong effect on the health of the individual. However, various factors can disrupt the development of the gut microbiota, leading to a condition known as dysbiosis, particularly in preterm infants. Current studies involving adults have suggested that the gut microbiota not only influences the gut but also has multidimensional effects on remote organs; these pathways are often referred to as the gut–organ axis. Imbalance of the gut microbiota may lead to the development of multiple diseases. Recent studies have revealed that gut dysbiosis in preterm infants may cause several acute morbidities—such as necrotizing enterocolitis, late‐onset sepsis, bronchopulmonary dysplasia, and retinopathy of prematurity—and it may also influence long‐term outcomes including neurodevelopment and somatic growth. This review mainly presents the existing evidence regarding the relationships between the gut microbiota and these morbidities in preterm infants and explores the role of the gut–organ axis in these morbidities. This paper thus offers insights into the future perspectives on microbiota interventions for promoting the health of preterm infants.

## 1 INTRODUCTION

The human gut is colonized by trillions of microbes, including bacteria, archaea, viruses, and unicellular eukaryotes; these microbes in the gut are collectively referred to as the gut microbiota.^1^ A symbiotic relationship is present between this microbiota and the gut, playing a crucial role in the maintenance of gut homeostasis, assisting in the maturation of gut function and structure, facilitating nutrition and metabolism, providing antimicrobial protection, and modulating the immune system.^2^ In neonates, the gut is quickly colonized by bacteria after birth, and the gut microbiota significantly develops in the first few years of life. Initially, he gut microbiota is low diversity and mainly composed of Proteobacteria and Actinobacteria, until the age of 2–3 years, the gut microbiota shifts toward an adult‐like composition, with Firmicutes as the dominant phylum.^3^ However, the composition of the gut microbiota can be disturbed by various internal and external factors, leading to a condition known as dysbiosis.^4^ This condition is especially common in preterm infants, and temporal dynamics and time‐dependent microbial shifts during gut microbiota development are observed in preterm infants; these dynamics are primarily influenced by age, postmenstrual age (PMA), and, to a lesser extent, gestational age.^5^ Other factors—including antenatal and postnatal antibiotic exposure, neonatal intensive care unit (NICU) environment, therapeutic intervention, feeding method, and mode of delivery—also contribute to an increased risk of dysbiosis.^4^, ^6^

**FIGURE 1:**
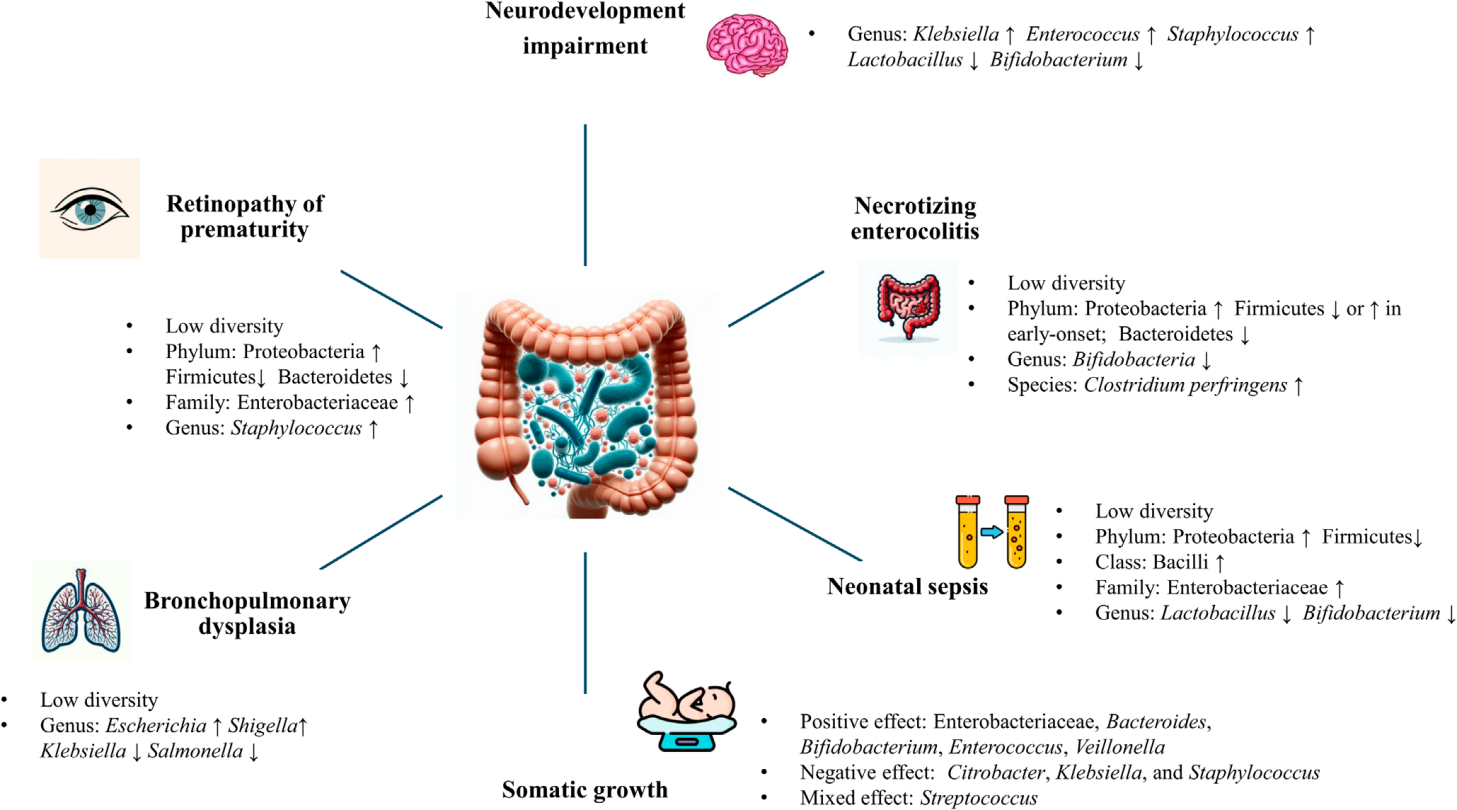
Gut microbiota alterations influence the development of morbidities in preterm infants.

Related studies involving adults have suggested that the gut microbiota not only affects gut diseases but also has a bi‐ or multi‐directional impact on multiple diseases affecting other organs. These pathways are often referred to as the gut–organ axis, which includes the gut–brain axis (GBA), gut–lung axis, gut–liver axis, gut–kidney axis, gut–heart axis, gut–skin axis, and gut–adipose axis.^7^ The interaction between the host and the gut microbes is mediated by multiple signaling pathways or chemical interactions, which can occur through nerve pathways or portal circulation or directly through the intestinal epithelial barrier into the bloodstream.^7^, ^8^ Similar to that in adults, gut dysbiosis in preterm infants may lead to several acute morbidities, including necrotizing enterocolitis (NEC), late‐onset sepsis (LOS), bronchopulmonary dysplasia (BPD), and retinopathy of prematurity (ROP). Gut dysbiosis may also affect long‐term outcomes, including neurodevelopment and somatic growth (Figure 1). This review mainly presents the existing evidence regarding the relationships between the gut microbiota and the aforementioned morbidities in preterm infants.

## 2 MORBIDITIES INFLUENCED BY THE GUT MICROBIOTA

### 2.1 Necrotizing enterocolitis

Although its pathogenesis is not fully understood, NEC is believed to result from a combination of factors, including genetic susceptibility, immature intestinal and immune systems, and inappropriate microbial colonization.^9^, ^10^ The hypothesis that microbial colonization plays a role in NEC development is supported by evidence indicating that NEC cannot be established in germ‐free animals^11^ and that concomitant bacteremia and endotoxemia are commonly observed in infants with NEC.^12^, ^13^ Furthermore, previous experiments utilizing cultures have identified that early colonization with *Clostridium perfringens* is a risk factor for NEC.^14^, ^15^, ^16^ With advanced high‐throughput molecular techniques for analyzing microbial DNA and RNA, recent evidence suggests that host–microbiota interactions play a major role in NEC development. Microbial dysbiosis—followed by a loss of barrier integrity, inflammation, and necrosis—may lead to NEC development. Rather than a single specific organism, aberrant interactions within the entire microbial community contribute to NEC development.^17^ In infants with NEC, Mai et al.^18^ found an increase of 34% in Proteobacteria bacteria and a decrease of 32% in Firmicutes bacteria between the 1‐week and <72‐h samples before the diagnosis of NEC. Subsequent studies have corroborated this increase in Proteobacteria bacteria and decrease in Firmicutes bacteria in the gut microbiota before the onset of NEC.^19^, ^20^, ^21^, ^22^ Notably, distinct aberrant microbial communities in the gut may contribute to variations in the timing of NEC onset.^23^, ^24^ Morrow et al.^23^ discovered that infants with NEC exhibited low alpha diversity and lacked *Propionibacterium* bacteria in their gut microbiota before the onset of NEC. In addition, early‐onset NEC (7–21 days after birth) has been observed to be associated with Firmicutes dysbiosis, whereas late‐onset NEC (19–30 days after birth) is associated with Proteobacteria dysbiosis.^23^ Zhou et al. reported similar findings, namely that *Clostridium* sensu stricto (from the Firmicutes phylum) bacteria were significantly high in quantity in infants with early‐onset NEC. Additionally, before late‐onset NEC, an increasing trend was found for Gammaproteobacteria (from the Proteobacteria phylum), including *Escherichia/Shigella* and *Cronobacter*, compared with controls.^24^ Stewart et al.^22^, ^25^ reported low microbial diversity to be a risk factor for NEC. Additionally, a decrease in Bacteroidetes and a low abundance of *Bifidobacteria* have been consistently reported as risk factors for NEC.^20^, ^22^, ^26^

### 2.2 Neonatal sepsis

Recent studies have demonstrated an association between the gut microbiota and neonatal sepsis. Madan et al.^27^ found that gut microbial patterns may predict the onset of neonatal sepsis; they observed that preterm infants with low microbial diversity at birth were at risk of LOS and that those with a predominance of *Staphylococcus* in their gut microbiota were likely to develop LOS. Mai et al. discovered that in the 2 weeks preceding LOS development in preterm infants, the gut microbiota was characterized by low diversity, an increasing trend for the Proteobacteria phylum, and a decreasing trend for the Firmicutes phylum. Furthermore, *Bifidobacteria* counts were consistently low in infants with LOS.^28^ In case–control studies conducted in two hospitals, Taft et al. observed that the characteristics of the gut microbiota before LOS onset varied by location. In Cincinnati, United States, infants who would later develop LOS exhibited lower *Actinobacteria* counts after birth and lower levels of *Pseudomonadales*. In Birmingham, United States, infants who would later develop LOS exhibited lower microbial diversity and lower levels of *Lactobacillales*.^29^ Shaw et al.^30^ observed an abundance of *Staphylococci* and Enterobacteriaceae in the gut microbiota of preterm infants with LOS in the week before the LOS onset. Stewart et al. observed that before LOS onset, the genus of the causative pathogen was prevalent in the gut microbiota. They also noted that the relative abundance of *Bifidobacterium* could provide protection against LOS development.^26^ Liu et al.^31^ reported that before the onset of NEC and LOS, preterm infants exhibited higher levels of *Bacillus* and *Solibacillus*. Graspeuntner et al.^32^ discovered that preterm infants with LOS exhibited a predominance of *Bacilli* and low diversity in their gut microbiota. Lee et al.^33^ found that the gut microbiota of infants with LOS was characterized by low diversity at birth as well as abnormal evolution with an increase in the Proteobacteria phylum and a decrease in the Firmicutes phylum. In summary, several characteristics of the gut microbiota—such as low diversity, overcolonization by pathogenic aerobes or facultative anaerobes, undercolonization by *Bifidobacterium*, and a disruption in the evolution of the microbiota from Proteobacteria dominance to Firmicutes dominance—are associated with the risk of neonatal sepsis.^3^

### 2.3 Nutrients and somatic growth in preterm infants

Altered gut microbiota and their metabolites directly influence nutrient usage, intestinal development, inflammation, and hormonal signaling, thereby affecting the growth and metabolic disorders in preterm infants.^34^, ^35^, ^36^ Nutrition significantly influences the development of the neonatal gut microbiota by facilitating essential diet–microbial interactions. Nondigestible substrates, such as human milk oligosaccharides (HMOs), are fermented by gut microbes, generating energy and absorbable growth‐promoting metabolites such as short‐chain fatty acids (SCFAs), including butyrate, acetate, lactate, and indole‐3 lactic acid.^37^ Butyrate produced through microbial metabolism induces T‐regulatory cell expression in the intestine and bone marrow, promoting bone formation.^38^ Gut microbes also influence the somatotropic axis that acetate deficiency potentially impairs insulin/insulin‐like growth factor (IGF) signaling.^34^ Lactate‐producing bacteria such as *Bifidobacterium* and *Lactobacillus* aid in gut colonization, enhancing the maturation of epithelial and mucosal dendritic cells. HMO fermentation produces indole‐3 lactic acid,^39^ an anti‐inflammatory metabolite that can promote growth in infants and reduce the risk of bacterial translocation by enhancing the expression of tight junction proteins in colonocytes.^40^ These findings highlight the pivotal role of nutrition in shaping the neonatal gut microbiota, which is crucial for growth and immune development.

Despite improvements in modern nutrition, postnatal growth failure frequently occurs in premature infants; a previous study found growth failure in up to half of a sample of infants in the <10th percentile of weight at discharge.^41^ The gut microbiota, particularly *Lactobacillus* abundance in the meconium, is correlated with the growth trajectory of preterm infants aged up to 10 months.^42^ Unique microbial patterns in early life (less than 3 months of age) may restrict later growth in children aged 2–5 years.^43^ Interactions between *Bacteroides* and *Enterobacter* at birth influence the growth of preterm infants; this finding offers insights related to fostering healthier growth for such infants.^43^ Neves et al.^44^ conducted a systematic review of the relationship between the gut microbiota and early postnatal growth, and they concluded that the neonatal gut microbiota can be positively or negatively associated with early postnatal growth. For instance, Enterobacteriaceae, *Bacteroides*, *Bifidobacterium*, *Enterococcus*, and *Veillonella* were all positively associated with growth, whereas *Citrobacter*, *Klebsiella*, and *Staphylococcus* were all negatively associated with growth. Across studies, mixed associations have been found between *Streptococcus* and growth.^44^

## 3 MORBIDITIES INFLUENCED BY THE GUT MICROBIOTA THROUGH THE GUT–ORGAN AXIS

### 3.1 The GBA and neurodevelopmental outcomes

Bidirectional mechanisms underlie the early developmental connections between the gut and brain. Brain signals influence gut functions, and gut‐derived molecules influence various processes in the brain through neural, endocrine, immune, and metabolic pathways.^45^ The GBA is crucial for mediating the interdependency of the enteric nervous system (ENS) with the central nervous system (CNS) and the autonomic nervous system (ANS). The ENS receives inputs from the brain and transmits information through ascending neural circuits. The ANS, comprising sympathetic and parasympathetic nerves, modulates gut function. This modulation occurs through the detection of the vagus nerve and the transmission of signals from the gut to the brain, and the ANS also interfaces with the limbic system to regulate various processes in the brain.^45^ Humoral components are involved in the hypothalamic–pituitary–adrenal axis, enteroendocrine system, and immune system.^46^ Microglia, the resident immune cells of the brain, regulate neurogenesis and synaptogenesis within the GBA. Their maturation and function are linked to alterations in the gut microbiota and are influenced by SCFAs.^47^ Immune signaling molecules produced by the gut microbiota can affect the GBA by binding to vagus nerve receptors or by crossing the blood–brain barrier.^48^ Metabolic mediators such as serotonin and SCFAs play crucial roles in gut–brain signaling, intestinal mucosal immunity, and systemic inflammation, thereby influencing neuroinflammation in the CNS.^49^, ^50^

Few studies have explored the relationship between the gut microbiota in preterm infants and their subsequent neurodevelopment, suggesting that associations between microbial dynamics in early infancy and subsequent neurodevelopmental outcomes may exist.^51^ For instance, Seki et al.^52^ investigated 60 extremely preterm infants and found that *Klebsiella* overcolonization at 6 weeks of age predicted brain damage before NICU discharge and that *Klebsiella* overcolonization was accompanied by the secretion of proinflammatory cytokines and reduced levels of neuroprotectants. Dysbiosis, which is characterized by reduced levels of *Bifidobacteria* and *Lactobacilli* and increased levels of pathogenic bacteria, precedes the development of NEC and LOS, which are associated with increased mortality and adverse effects on growth and neurodevelopment.^21^, ^33^ The EPIFLORE cohort study identified six bacterial patterns for the gut microbiota at 4 weeks after birth; perinatal and postnatal factors contributed to these patterns, and *Enterococcus* and *Staphylococcus* clusters were significantly associated with adverse outcomes at the age of 2 years.^53^ Similarly, Beghetti et al.^54^ found that the gut microbiota of very‐low‐birth‐weight infants in the first month was correlated with neurodevelopmental outcomes at the age of 24 months and that the absence of *Bifidobacterium* at 30 days was associated with neurodevelopmental impairment in early childhood. In addition, recent systematic reviews have revealed an association between the gut microbiota of infants and neurodevelopmental outcomes in preterm infants.^51^, ^55^ Nevertheless, inconsistencies exist in microbial patterns and characteristics with respect to time, assessment timing, and neurodevelopmental evaluation methods across studies, thereby limiting the understanding of the GBA in preterm infants. Moreover, exploring the impact of clinical variables (e.g., LOS, NEC, and feeding type) and expanding the time window for microbiota analysis beyond early infancy are crucial for advancing research in this area.

### 3.2 The gut–lung axis and bronchopulmonary dysplasia

In newborn infants, especially intubated preterm infants, the microbiota in the airway is established soon after birth.^56^, ^57^ Recent studies have demonstrated an association between the lung microbiota and BPD in preterm infants. Lohmann et al. discovered that newborns who subsequently developed BPD had low microbial diversity at the time of intubation compared with those who had no BPD; that study also observed changes in the lung microbiota of infants with BPD, with an increase in Firmicutes bacteria and a decrease in Proteobacteria bacteria, contrasting with the diverse and stable microbial community in the non‐BPD group. Although *Acinetobacter* was the dominant genus in both groups, its relative abundance decreased over time in the BPD group, whereas the levels of *Staphylococcus* and *Klebsiella* increased.^57^ However, the findings regarding the relationship between dysbiosis and BPD are inconsistent. Lal et al. reported an increase in the levels of the Proteobacteria phylum and a decrease in the levels of the Firmicutes and Fusobacteria phyla in the lung microbiota, which was associated with BPD diagnosis. Comparing extremely‐low‐birth‐weight (ELBW) infants and term infants, at the phylum level, the lung microbiota predominantly consisted of Proteobacteria and Firmicutes in both groups, with the exception of an abundance of *Ureaplasma* in the ELBW group.^58^ Wagner et al.^59^ reported that preterm infants with severe BPD exhibited greater bacterial community turnover with age and that these infants acquired fewer initial *Staphylococcus* bacteria and more *Ureaplasma* bacteria in the first few days after birth. Finally, Imamura et al.^60^ found that higher levels of *Corynebacterium* species were detected in infants with severe BPD than in those without it.

The gut microbiota may influence the development of airway diseases through mechanisms involving the reciprocal exchange of microbes and their metabolites as well as immunomodulatory signals between the gut and lungs, known as the gut–lung axis.^61^, ^62^ The gut microbiota can influence pulmonary function. This influence is exerted by the production of ligands, metabolites, and immune cells that are transported to the lungs through the bloodstream, thereby regulating pulmonary immunity. Through these circulating cells and metabolites, the gut microbiota may directly affect pulmonary immunity, thereby potentially altering the composition of the lung microbiota.^63^ In preterm infants, associations between gut dysbiosis and BPD have been reported. Ryan et al.^64^ observed that in infants born vaginally who developed BPD, an increased abundance of *Escherichia* and *Shigella* and a decreased abundance of *Klebsiella* and *Salmonella* were found in their the gut microbiota compared with infants without BPD. Additionally, Chen et al.^65^ discovered that compared with infants without BPD, infants with BPD exhibited significantly low diversity in their gut microbiota from birth. However, studies have also suggested that lower lung microbiota is more likely to influence the severity of BPD than is the gut microbiota.^56^, ^66^ This finding implies that the gut microbiota may affect BPD indirectly, such as through the gut–lung axis. The gut microbiota may activate immune pathways, such as NOD‐2 and IL‐17A signaling through the granulocyte‐macrophage colony‐stimulating factor signaling pathway, which increases the activity of alveolar innate macrophages for capturing pathogens (especially strong NOD‐2 activators such as *Lactobacillus reuteri*, *Enterococcus faecalis*, *Lactobacillus crispatus*, and *Clostridium orbiscindens*), thereby preventing pulmonary infection.^67^ Because of decreased pulmonary immunity, recurrent infection may trigger the production of interleukin (IL)‐1β, IL‐6, IL‐8, NLRP3, tumor necrosis factor‐α, and collagen I, which in turn may reduce the levels of surfactant and vascular growth factors.^68^ In addition to proinflammatory cytokines, gut and lung dysbiosis promotes IL‐17B secretion by Th‐17, which is related to lung fibrosis.^69^, ^70^ Further research is required to understand how the gut microbiota influences the development of BPD and the role of the gut–lung axis in preterm infants.

### 3.3 The gut–retina axis and retinopathy of prematurity

ROP is a vasoproliferative disorder of the retina occurring in preterm infants.^71^ It is a leading cause of visual impairment and blindness in children, and the global prevalence of ROP 31.9% among all preterm infants.^72^ Low gestational age and low birth weight are well‐established risk factors. However, its etiology is considered multifactorial, with prolonged oxygen treatment, mechanical ventilation, perinatal infection, systemic inflammation, and genetic factors have been widely investigated.^73^, ^74^ Emerging evidence suggests that the neonatal gut microbiota plays a role in the development of ROP.^75^ Studies have found distinct gut microbial compositions in preterm infants with ROP compared with those without ROP. For instance, Skondra et al.^75^ observed a significantly high abundance of Enterobacteriaceae at 28 weeks PMA in infants who later developed type 1 ROP. These infants also exhibited decreased alpha diversity. Similarly, Westaway et al.^76^ assessed probiotic‐treated premature infants, and the results revealed that compared with the corresponding levels at admission, significantly reduced alpha diversity of the gut microbiota and significantly abundant *Staphylococcus* were found at the time of discharge in those who developed ROP.

The concept of the gut–retina axis has been supported by studies linking gut dysbiosis to retinal pathologies.^77^ Gut dysbiosis can lead to increased intestinal permeability and systemic inflammation.^78^ The association between gut dysbiosis and neonatal comorbidities has provided an insight into the pathogenesis of ROP. In preterm infants who developed NEC, a condition associated with gut dysbiosis, the dominance of the Enterobacteriaceae family in the gut microbiota was noted.^79^ In addition, infants with early surgical NEC were at significantly increased risk of ROP.^80^ Given the association between NEC and ROP, it is plausible that gut dysbiosis may contribute to the development of ROP. This link could be explained by the dysregulation of vascular endothelial growth factor (VEGF) and IGF‐1.^81^ For example, pathogenic bacteria such as *Escherichia coli* have been shown to upregulate levels of VEGF, potentially driving the neovascularization observed in ROP.^82^ In animal models, increased *Proteobacteria* counts and decreased Firmicutes and Bacteroidetes counts were related to a significant reduction in serum IGF‐1.^83^ IGF‐1 is crucial for retinal vascular development, and its deficiency is linked to ROP severity. In addition, immune dysregulation and systemic inflammatory responses can exacerbate retinal oxidative stress and lead to subsequent ROP progression.

A few studies have identified changes in the gut microbiota and the metabolic profile. For example, one study found that excessive arginine metabolism was associated with increased oxidative stress in retinal cells. Compared with controls, ROP patients exhibited significantly elevated plasma levels of citrulline, arginine, and aminoadipic acid.^84^ In an oxygen‐induced retinopathy rat model, Lu et al.^85^ discovered that alternating hyperoxia/hypoxia conditions induced significant changes in the richness of proline, ornithine, and glutamine, all of which are crucial for the metabolism of arginine and proline. Given these associations, several clinical and preclinical studies have assessed potential therapeutic targets for ROP, such as the infusion of recombinant human IGF‐1, probiotics, and SCFA supplementation.^86^ However, most findings to date have been associative, and thus further research is needed to establish the causative mechanisms and to elucidate the precise effects of these therapies on ROP.

## 4 FUTURE PERSPECTIVES ON MODULATING THE GUT MICROBIOTA IN PRETERM INFANTS

Given that preterm infants are suspectable to dysbiosis of the gut microbiota and that such dysbiosis is linked to adverse morbidities and outcomes, early interventions to modulate the gut microbiota are promising for enhancing the health of preterm infants. Potential treatment strategies include the modulation of microbiota through the administration of prebiotics, probiotics, or synbiotics, as well as the implementation of treatments for dysbiosis, such as fecal microbiota transplantation (FMT). FMT, which involves the transfer of fecal material from a healthy individual to a person with gut dysbiosis to restore their dysbiotic microbiota, has been increasingly utilized for the treatment of recurrent *Clostridioides difficile* infection and other gastrointestinal disorders.^87^ However, currently, clinical research regarding FMT use in preterm infants is lacking, and the use of FMT to prevent or reverse damage caused by NEC is still being validated in animal models.^88^, ^89^ In a human study, a proof‐of‐concept investigation demonstrated that maternal FMT in infants born through cesarean section successfully led to typical gut microbiota development, constituting a promising outcome in the field of microbial engineering.^90^ However, the clinical application of FMT in preterm infants requires thorough evaluation and research into its safety profile given that FMT carries the potential risk of transmitting pathogens, including multidrug‐resistant bacteria and antimicrobial resistance genes.^87^

Currently, evidence suggests that the gut microbiota of preterm infants can be modulated by supplementation with probiotics.^91^ The oral supplementation of preterm infants with probiotics containing *Bifidobacterium* and *Lactobacillus* led to an increase in probiotic bacterial strains and inhibited the growth of potentially pathogenic bacteria in the gut microbiota.^92^, ^93^ Additionally, the gut microbiota of preterm infants supplemented with probiotics was highly similar to that of term infants of the same age by the time the preterm infants reached term age.^94^ Increasing evidence supports the idea that the administration of probiotics may alter an individual's microbial composition and thus can provide benefits for preterm infants. Most related systematic reviews and meta‐analyses, whether observational or randomized case–control studies, have demonstrated that probiotics may substantially reduce the risks of LOS and NEC and the mortality rate of preterm infants.^95^, ^96^, ^97^ However, the currently available evidence is insufficient for determining whether probiotics can mitigate the risks of other morbidities, such as BPD and ROP, and improve growth and neurodevelopmental outcomes.^98^, ^99^

## 5 CONCLUSIONS

Preterm infants are particularly susceptible to the abnormal development of gut microbiota and vital organs. Although links have been established between gut dysbiosis and morbidities, further investigations regarding the correlations of specific bacterial taxa with specific outcomes and the variable effects of microbial interventions are warranted. The mechanisms underlying the distinct effects of the combinations of specific taxa should be determined to refine the application of prebiotics and probiotics, thereby reducing morbidity risks and improving outcomes for preterm infants. Current related research gaps include a lack of standardized protocols for probiotic regimens, dosing, and treatment duration. Moreover, the role of the gut microbiota in disease processes through the gut–organ axis should be explored and clarified. Finally future research should aim to tailor microbiota engineering interventions and establish clinical guidelines for ensuring the healthy development of the gut microbiota, which would likely ultimately improve health outcomes for preterm infants.

## CONFLICT OF INTEREST STATEMENT

All authors declare that there are no conflicts of interest.

## NEONATAL MICROBIOME OUTCOMES STUDY GROUP (NEMO)

The members of the NEMO Study Group include: Cheng‐Hsun Chiu, MD PhD (Division of Pediatric Infectious Diseases, Department of Pediatrics, Chang Gung Memorial Hospital); Ming‐Chou Chiang, MD (Division of Neonatology, Department of Pediatrics, Chang Gung Memorial Hospital); Wei‐Chi Wu, MD, PhD (Department of Ophthalmology, Chang Gung Memorial Hospital); Chien‐Chung Lee, MD, PhD (Division of Neonatology, Department of Pediatrics, Chang Gung Memorial Hospital); Yuan‐Ming Yeh, PhD (Genomic Medicine Core Laboratory, Chang Gung Memorial Hospital); Mei‐Yin Lai, MD (Division of Neonatology, Department of Pediatrics, Chang Gung Memorial Hospital); Wei‐Hung Wu, MD (Division of Neonatology, Department of Pediatrics, Chang Gung Memorial Hospital); Yin‐Hsi Chang, MD (Department of Ophthalmology, Chang Gung Memorial Hospital); Po‐Yi Wu, MD (Department of Ophthalmology, Chang Gung Memorial Hospital); Ming‐Chih Ho, MD (Department of Ophthalmology, Chang Gung Memorial Hospital).
